# The Cooperative Health Research in South Tyrol (CHRIS) study: rationale, objectives, and preliminary results

**DOI:** 10.1186/s12967-015-0704-9

**Published:** 2015-11-05

**Authors:** Cristian Pattaro, Martin Gögele, Deborah Mascalzoni, Roberto Melotti, Christine Schwienbacher, Alessandro De Grandi, Luisa Foco, Yuri D’Elia, Barbara Linder, Christian Fuchsberger, Cosetta Minelli, Clemens Egger, Lisa S. Kofink, Stefano Zanigni, Torsten Schäfer, Maurizio F. Facheris, Sigurður V. Smárason, Alessandra Rossini, Andrew A. Hicks, Helmuth Weiss, Peter P. Pramstaller

**Affiliations:** Center for Biomedicine, European Academy of Bolzano/Bozen (EURAC) (Affiliated to the University of Lübeck, Lübeck, Germany), Via Galvani 31, 39100 Bolzano/Bozen, Italy; Respiratory Epidemiology, Occupational Medicine and Public Health, National Heart and Lung Institute, Imperial College, London, UK; Functional MR Unit, Policlinico S. Orsola, Malpighi Bologna, Department of Biomedical and Neuromotor Sciences, University of Bologna, Bologna, Italy; Dermatological Practice, Kirchplatz 3, 87059 Immenstadt, Germany; The Michael J. Fox Foundation for Parkinson’s Research, New York, NY, USA; Hospital of Schlanders/Silandro, Schlanders/Silandro, Italy; Department of Neurology, Central Hospital, Bolzano, Italy; Department of Neurology, University of Lübeck, Lübeck, Germany

## Abstract

The Cooperative Health Research In South Tyrol (CHRIS) study is a population-based study with a longitudinal lookout to investigate the genetic and molecular basis of age-related common chronic conditions and their interaction with life style and environment in the general population. All adults of the middle and upper Vinschgau/Val Venosta are invited, while 10,000 participants are anticipated by mid-2017. Family participation is encouraged for complete pedigree reconstruction and disease inheritance mapping. After a pilot study on the compliance with a paperless assessment mode, computer-assisted interviews have been implemented to screen for conditions of the cardiovascular, endocrine, metabolic, genitourinary, nervous, behavioral, and cognitive system. Fat intake, cardiac health, and tremor are assessed instrumentally. Nutrient intake, physical activity, and life-course smoking are measured semi-quantitatively. Participants are phenotyped for 73 blood and urine parameters and 60 aliquots per participant are biobanked (cryo-preserved urine, DNA, and whole and fractionated blood). Through liquid-chromatography mass-spectrometry analysis, metabolite profiling of the mitochondrial function is assessed. Samples are genotyped on 1 million variants with the Illumina HumanOmniExpressExome array and the first data release including 4570 fully phenotyped and genotyped samples is now available for analysis. Participants’ follow-up is foreseen 6 years after the first visit. The target population is characterized by long-term social stability and homogeneous environment which should both favor the identification of enriched genetic variants. The CHRIS cohort is a valuable resource to assess the contribution of genomics, metabolomics, and environmental factors to human health and disease. It is awaited that this will result in the identification of novel molecular targets for disease prevention and treatment.

## Background

The Cooperative Health Research In South Tyrol (CHRIS) study is a population-based study with a longitudinal lookout established in 2011 to investigate the genetic basis of common chronic conditions associated with human ageing, and their interaction with life-style and environmental factors in the general population of South Tyrol. Located in the middle of the Alpine mountainous region, the landscape is characterized by rural and small villages across many valleys. Agriculture and tourism are the major drivers of the gross domestic product of the region, which is among the highest in Europe. In a context of a general worldwide life expectancy increase, ageing is expected to be longest in such high income regions [1], which will also be characterized by a relevant demographic deficit [2], as documented in a recent report from the Autonomous Province of Bolzano/South Tyrol [3].

The CHRIS study is focused on cardiovascular, metabolic, neurological, and psychiatric health. Cardiovascular and metabolic diseases are major components of all non-communicable diseases, whose burden will continue to increase over the next decades [1]. By 2030, more than 40 % of the adult population could be affected by at least one cardiovascular condition [4]. Cardiovascular diseases (CVD) will become the leading cause of death in 65 + -year-old subjects and, at the current ageing rate, the cost to treat CVD will triplicate [5]. Similarly, prevalence of metabolic syndrome [6], diabetes, and particularly prediabetes [7], is rising. Among the neurological conditions, the most prevalent disease in European regions of low child and adult mortality is migraine [8]. However, the increased burden of neurological diseases over the next fifteen years will be mainly due to the rising prevalence of dementias [9] and neuropathies. The CHRIS study pays particular attention to neurodegenerative movement disorders, since they are generally under-recognized and under-treated, with a relevant impact on life quality and health economy. A South Tyrolean study representative of the general population [10] reported a prevalence of ~ 28 % in 50–89-year-old subjects, sharply increasing with age. Psychiatric health is also a major concern of European countries, with 14 % of the population suffering of anxiety disorders and nearly 7 % of major depression, with no particular differences by country [11].

Biomedical research is needed to identify factors that affect the ageing process, which may lead to preventive interventions for healthy ageing with reduction of health care related costs. For this reason, the CHRIS study was established as a collaboration between a research institute (the EURAC Center for Biomedicine) and the South Tyrolean Health System. Such a collaboration guarantees that the study operates by actively interacting with the local population, thus raising awareness towards a more conscious approach to health. The study is expected to foster a dynamic cycle among scientists, clinicians, and the whole population, which is to offer reciprocal feedback to ultimately improve individuals’ health.

## Methods

### Design, reference population, and recruitment

#### Study objectives

The CHRIS study has been established to accomplish the following objectives: (1) to identify biological mechanisms underlying cardiovascular, metabolic, neurological, and psychiatric health, and to understand how these mechanisms can be influenced by environmental exposures; (2) to raise population’s awareness towards prevention and health.

#### Study design and reference population

**Fig. 1:**
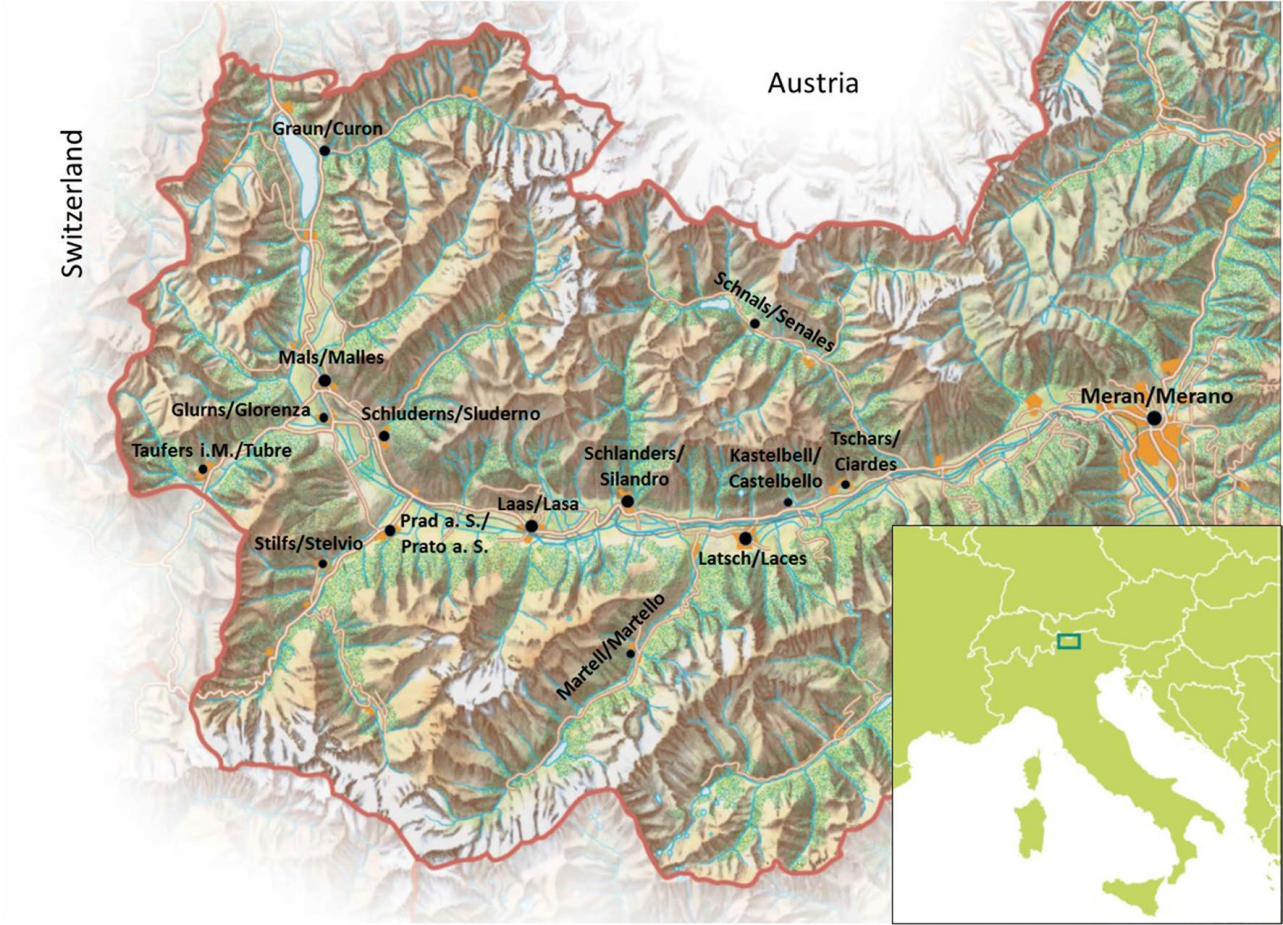
Geography of the CHRIS study. Indicated are all municipalities involved in the study and the biobank location (Meran/Merano). Map source: Südtirol Marketing

The CHRIS study invites all 18+-year-old inhabitants of the middle and upper Vinschgau/Val Venosta, a 70 km long valley in the autonomous province of Bolzano/South Tyrol, Italy, at the border with Switzerland and Austria (mother tongue: German 97 %, Italian 3 %) [12]. The area comprises 13 municipalities, each one characterized by a main center (inhabitants 270–4300), about 30 sparse villages (inhabitants 50–800), and scattered mountain farms (Fig. 1). All settlements are located at an altitude of about 600–2000 m above sea level. At the time when the study started (24 Aug 2011), 28,497 adult residents were registered in the electoral lists and formed our sampling basis. The aim is to recruit at least 10,000 participants, roughly corresponding to 35 % of the whole adult population. To date, over 7000 adults already participated. Completion of the first phase of the study is expected by mid-2017.

#### Longitudinal perspective of the CHRIS study and follow-up of the MICROS study

The study has a longitudinal design plan, with follow-up starting after 6 years from initial recruitment. In addition, the CHRIS study is being conducted in the same geographical area where the MICROS study was previously carried out in 2002/03 [12]. The MICROS study involved three of the municipalities included in the CHRIS study. All former living MICROS participants still resident in the area are now being invited into the CHRIS study. This group consists of 1259 subjects, for whom DNA and extensive clinical and biological phenotypes [13, 14] are available. With a 10+ year follow-up, those accepting to re-participate will thus constitute the first longitudinal nucleus within the CHRIS study.

#### Recruitment strategy

**Fig. 2:**
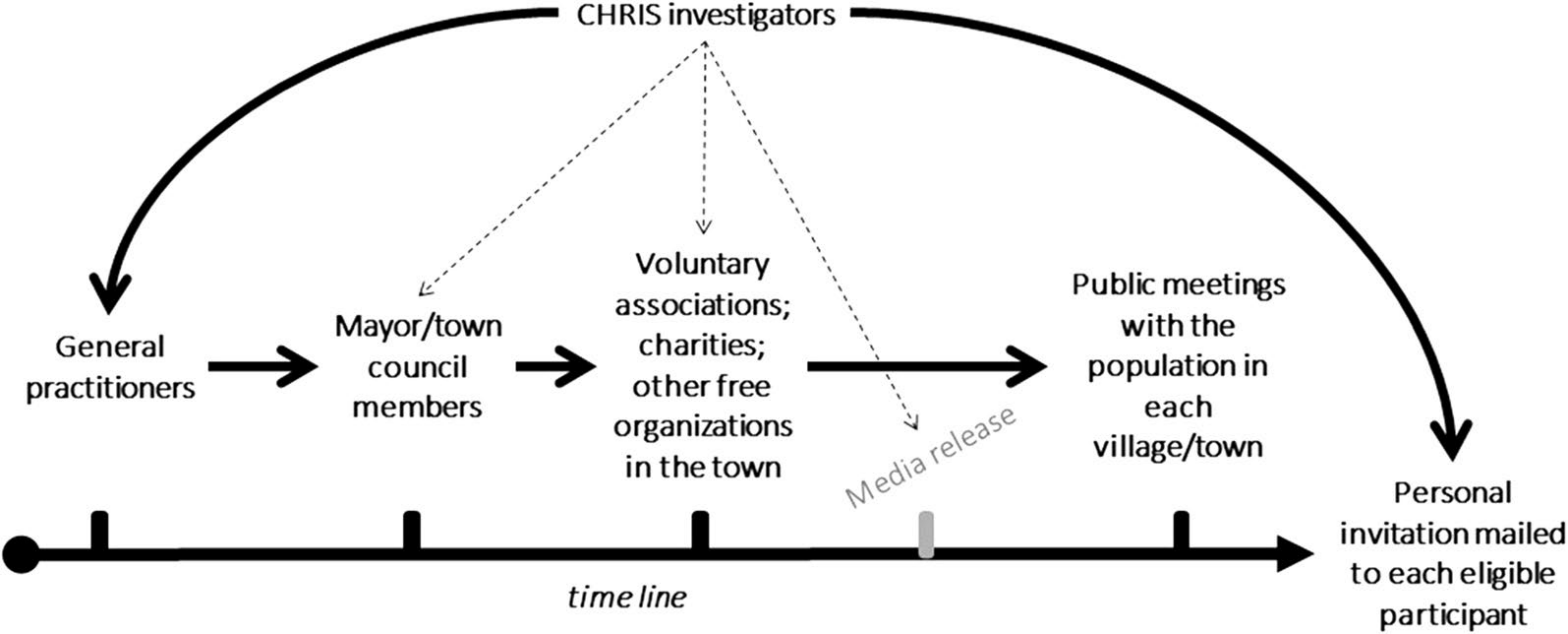
Communication workflow

The recruitment center is located in the valleys’ reference hospital of Schlanders/Silandro, the central town of the valley. Population involvement proceeds gradually, expanding the study from one municipality to the contiguous one. This design allows a tailored communication strategy and monitoring of participation rate. In each municipality, the recruitment begins after an informative campaign (Fig. 2): (1) CHRIS study investigators share the study concept with local general practitioners (GPs); (2) GPs and CHRIS investigators meet the mayor and other relevant members of the municipality council; (3) the municipality council invites all leaders of local charities and voluntary organizations to a meeting with CHRIS investigators; (4) the study is first announced to the population through the local media and then the entire population is invited to one or more public meetings where the study is officially introduced to the community. This last step guarantees direct interaction and discussion with the public. Afterwards, a first invitation letter is mailed to all 18+-year-old inhabitants, who are identified through publicly available electoral lists. To favor the identification of genetic variants that might be enriched in single families, we encourage participation of the entire family. For this reason, the first invitation is mailed personally to each member of the same family at the same time. Up to two reminders are mailed to non-responders to maximize participation into the study. All communication is bilingual (German/Italian) to equally involve both linguistic groups.

#### Enrollment and visit at the study center

The study is organized to enroll up to 10 participants/day, who are required to have fasted overnight. After the informed consent procedure, participants undergo tremor assessment, blood drawing, urine collection, anthropometric measurements, a 20-min electrocardiographic (ECG) analysis, and blood pressure measurement. Breakfast is offered after completion of all assessments to avoid any short-term effect of sugar, fat or caffeine consumption. Finally, participants respond to a computer assisted personal interview (CAPI) and a computer aided self-interview (CASI).

### Ethical and legal framework: a participant-centric approach

The CHRIS study was approved by the Ethical Committee of the Healthcare System of the Autonomous Province of Bolzano (Südtiroler Sanitätsbetrieb/Azienda Sanitaria dell’Alto Adige), protocol no. 21/2011 (19 Apr 2011). In addition, the CHRIS study invested in creating a comprehensive ethical, legal, and social implication (ELSI) framework aimed at building and ensuring long lasting trust and participation [15]. The study is compliant with current Italian and EU regulation and with the Helsinki Declaration. Privacy and security in data handling and sharing are strictly enforced and a public access code regulates how data and samples can be used. Data and samples are only shared for specific projects and based on Material and/or Data Transfer Agreements [16].

#### Dynamic informed consent

Given that the CHRIS study is designed to be longitudinal, with use of data and samples that will be extensive and prolonged in time, an interactive dynamic consent process for empowering participants’ autonomy and complying with current regulations was also implemented. Dynamic consent includes two important parts: an ongoing information section and an interactive consent webpage with dynamic options.

Information is provided in different formats to improve understanding through the use of diverse media that replace the information sheet. After booking the appointment, participants receive at home (by post or email) a detailed information brochure (http://www.chrisstudy.it), which includes a description of the study, images illustrating key concepts in lay language, and all ethical and legal issues relevant for the informed consent process. At the study center, the participant is invited to watch a 9 min information movie (available on the study webpage) that systematically and fully explains the project. The movie shows the whole research workflow, outlines how data and samples are handled, what security measures are in place and what are the risks involved, and it describes the participant’s rights and information sources through images and small animations. After viewing, the participant can ask questions to the study assistants. While it was not meant to replace the oral communication between participant and study assistant, the introduction of the movie had the effect of shortening the time needed for further explanations from about 20 min before its introduction to less than 5 min after its implementation. A yearly newsletter and updated information on the webpage complement the ongoing information for consent.

After the movie, electronic consent is filled in online directly on the personal interactive consent webpage. The type of consent asked is broad with regard to the aim of the study. At the same time, the consent is layered and provides dynamic options (changeable online over time) regarding data-sharing (international, public data repositories), return of secondary/unexpected results (outlining the right to know or the right not to know) and the permission to use samples and data in case of death. The data about the access levels granted by each participant goes directly into the database and is linked to their unique identification code. In this way data can be automatically filtered for different purposes according to participants’ choices. The dynamic tool can also be used for re-contact, collecting additional information and re-consent, should this be necessary in the future. Participants can withdraw at any time by contacting the study center or online through username/password protected access. Three options are allowed: (a) complete withdrawal with data cancellation and sample destruction; (b) continued use of data but no re-contact; (c) data and sample usage limited to already running studies. A study on a sample of 500 consecutive participants is being carried out to assess participants’ feeling about the dynamic informed consent and satisfaction with the information provided.

#### Compensation and result notification

No financial compensation or travel cost reimbursement is offered to support participation. Before leaving the study center, participants receive a dismissal letter with the results of the anthropometric, impedance, and blood pressure measurements. One week after, participants receive a letter with the complete results of their clinical assessments, including blood, urine, and 10-s ECG results validated by a clinician. Participants are invited to discuss the results with their GP. Laboratory life-threatening findings are followed up through an emergency protocol which, via the study coordinator and the reference GP, guarantees that the participant is alerted in the shortest possible time. A senior medical doctor and the emergency department of Schlanders/Silandro hospital are covering necessary immediate interventions due to serious cardiac issues occurring during the ECG or problems arising during blood drawing according to the study’s emergency protocols.

#### Secondary findings

Return of unexpected secondary or health threatening results is provided upon prior explicit participant’s request. In such a case, a multistage consent takes place so that the participant can effectively take a decision at the time when information is available, thus confirming if he/she truly wants such results back. In the event of genetic incidental findings, an agreement with the Health Care System genetic counseling unit ensures that participants are approached by a medical geneticist, who undertakes proper counseling before results are tested and confirmed.

#### Governance

CHRIS governance refers to an internal committee which monitors everyday issues (data and sample access, study management) and three oversight external bodies: the ethical board, the scientific board, and an evaluation committee that evaluates the project’s major changes and includes stakeholders from the local healthcare system and study participants. Legal and ethical issues are described in the Ethical and Legal regulation published on the study website.

### Data management

A dedicated and password protected network has been set up at the study center to secure data safety, integrity, and privacy. A local network connecting all electronic devices is linked to, and managed by, a central server, which is regularly monitored and backed up. Participants are assigned a barcode, which is especially useful to enter personal data correctly using the National Fiscal Code badge and to link all data and samples to the participant (unique barcode for all tubes, forms, and datasheets from the same participant). Web interfaces are driven by the open source software LimeSurvey (http://limesurvey.org) and connected using a PostgreSQL database.

### Biospecimens

#### Collection

Blood (49 ml) and urine (30 ml) samples are collected at the study center for laboratory analysis and biobanking (Table 1). Biomarkers analysed include all main cardiovascular and metabolic risk factors (including fibrinogen, C-reactive protein, and homocysteine), antinuclear antibodies (marker of autoimmune disease and other metabolic dysfunction), and markers of iron metabolism, coagulation, renal damage, thyroid, and liver function. Given the instability of some of the blood and urine parameters, including glucose and homocysteine, pre-analytical sample processing is performed immediately at the study center by trained nurses, to ensure reliability and accuracy of all the measurements. Collected samples are shipped daily to the CHRIS laboratory at the hospital of Meran/Merano (Fig. 1) at room temperature or at 4 °C, as appropriate. During transportation, sample temperature is monitored and recorded by means of electronic thermometers placed inside the transportation bags. A software has been developed which uploads temperature measurements automatically to a central server. An alert is sent out when a temperature curve falls outside the predefined control range.

**Table 1:** Biospecimens: collection tube and volume, material, parameters, and destination

Collection tube type	Volume collected (ml)	Assay material	Purpose			
			Blood/Urine Analyses	Biobank		
			Parameter	No. of Aliquots	Aliquot volume (µl)	Storage temp. (°C)
Serum gel + Clot Act. Venosafe VF108SAS	16	Serum	Antinuclear antibodies; Albumin; CRP; Calcium; Chlorine; Potassium; Magnesium; Sodium; Phosphorous; Ferritin; Iron; Transferrin; Creatinine; Uric acid; Cholesterol; HDL; LDL; Triglycerides; Lipase; Direct and total bilirubin; GGT; AST; GOT; ALT; GPT; ALP; Cortisol; Glucose; anti-TPO AB; TSH; FT4 and FT3 in case of abnormal TSH	15	220	–80
K2-EDTA Venosafe VF053SDK	15	EDTA plasma	Monocytes; Basophils; Neutrophils; Lymphocytes; Eosinophils; HCT; HGB; Mean Red Blood Cell; MCH; MCHC; MCV; MPV; RDW; PLT; RBC; WBC; HbA1c	10	220	–80
		EDTA buffy coat in RNA later	/	4	490	–80
		DNA	/	2	Variable	–20
Na3-Citrate Buffered Venosafe VF054SBCS07	14.5	Citrate plasma	Antithrombin; Fibrinogen; INR; PT; aPTT	10	220	–80
		Citrate buffy coat +DMSO	/	1	900	–190
		Citrate whole blood + DMSO	/	3	1000	–190
Gel + Li.Heparin. Venosafe VF054SAHLAS	3.5	Heparin plasma	Homocysteine	/	/	/
Vacutest U218007/U14930	30	Urine	pH; Glucose; Proteins; Haemoglobin; Ketone bodies; Bilirubin; Urobilinogen; Specific weight; Nitrites; Leukocytes-esterase; Leukocytes; Erythrocytes; Epithelial cells; Bacteria; Creatinine; Albumin; ACR	15	220	–80

*CRP* C-reactive protein, *HDL* high density lipoprotein, *LDL* low density lipoprotein, *GGT* gamma-glutamyl transferase, *AST* aspartate aminotransferase, *GOT* glutamic oxaloacetic transaminase, *ALT* alanine aminotransferase, *GPT* glutamate pyruvate transaminase, *ALP* alkaline phosphatase, *anti*-*TPO AB* anti-thyroid peroxidase antibodies, *TSH* thyroid-stimulating hormone reflex, *FT4* free thyroxine, *FT3* free triiodothyronine, *HCT* haematocrit, *HGB* haemoglobin, *MCH* mean cell haemoglobin, *MCHC* mean red blood cell haemoglobin concentration, *MCV* mean red blood cell volume, *MPV* mean platelet volume, *RDW* red blood cell distribution width, *PLT* platelet count, *RBC* red blood cell count, *WBC* white blood cell count, *HbA1c* glycated hemoglobin, *INR* international normalized ratio, *PT* prothrombin time, *aPTT* activated partial thromboplastin time, *ACR* albumin-to-creatinine ratio

#### Biobanking

About 28 ml of the collected blood and 3.3 ml of urine undergo cryopreservation (Table 1). Fractionated blood is aliquoted in small volumes in 2D-barcoded screw-cap vials (Thermo Scientific™ Nunc™ Bank-It™ Vial Systems) using a robotic liquid handler (Starlet, Hamilton Robotics, CH-7402 Bonaduz, GR, Switzerland). Biospecimens are flash frozen via direct immersion in liquid nitrogen and stored at −80 °C. Whole blood and buffy coat aliquots are stored at −190 °C in nitrogen vapor. To maintain cell vitality in those aliquots, 10 % dimethyl sulfoxide is added (Sigma-Aldrich, D2650) and freezing rate is controlled (CoolCell alcohol-free cell freezing containers, BioCision, CA, USA) until the samples reach −80 °C before transfer to −190 °C storage. Three milliliters of EDTA whole blood are separated, frozen at −20 °C, and sent to a second biobank in Bozen/Bolzano for DNA extraction. This second biobank works also as a safety backup and receives 50 % of the samples for storage. Genomic DNA extraction is performed using an automated platform for nucleic acid isolation (Chemagic Magnetic Separation Module I, PerkinElmer Chemagen Technologie GmbH, Germany) using a dedicated method based on magnetic beads (Chemagic DNA Blood Kit special, PerkinElmer Chemagen Technologie GmbH, Germany). DNA is automatically handled through a robotic liquid handler (Starlet, Hamilton Robotics, CH-7402 Bonaduz, GR, Switzerland), eluted in 300 μl, quantified through absorbance (Epoch, Take3 Trio Micro-Volume Plate, BioTek Instruments, USA), and stored at –20 °C. A child aliquot (100 μl volume) is normalized at 100 ng/μl and stored at 4 °C.

Sample management, operation, and monitoring instruments are integrated in a Biobank Information Management System (BIMS). The system allows the monitoring of all standardized procedures and sample-handling operations. Access to the bioresource is regulated through an access regulation for internal and external use. The biobank has joined the Biobanking and Biomolecular Resources Research Infrastructure (BBMRI) which provides protocols that guarantee top-level biological and medical research by promoting procedure standardization and sample quality. In order to maximize transparency on the use of samples and data and for tracking the use of the bioresource, the CHRIS biobank was assigned a “Bioresource Research Impact Factor” (BRIF) code (http://www.p3g.org/brif-bioshare-pilot-study): BRIF6107.

#### Genotyping

All CHRIS samples are genotyped on ~1 million single nucleotide polymorphisms (SNPs) with the Illumina HumanOmniExpressExome Bead Chip, which includes ~250,000 exonic variants. High coverage exome sequencing is foreseen for a large proportion of participants.

### Assessing the compliance with a paperless study

CHRIS is predominantly a paperless study. Interviews and data-collection procedures are computer aided. At the planning stage, a feasibility study was performed to compare two modes of interview: a computer-assisted self-administered (CQ) versus a paper-based self-administered (PQ) survey questionnaire. A convenience sample of 66 adults was prospectively recruited from a general practice in the same rural area of the CHRIS study. Questionnaires and individual questions were selected to represent a broad range of both sensitive domains and measurement scales [17–19]. Two independent groups completed a test–retest assessment of the same questionnaire (PQ–PQ, n = 17; and CQ–CQ, n = 14). Two further independent groups completed the survey on both platforms according to a random sequence (cross-over design; PQ-CQ, n = 16; CQ-PQ, n = 19). Questionnaire contents and structure were identical for the two platforms. The two platforms were compared according to measures of efficiency (e.g. completion time), concordance, and general appraisal. In the cross-over group, concordance was excellent, with intraclass correlation coefficients ranging between 0.88 (Center for Epidemiologic Studies of the National Institute of Mental Health Depression scale [18], CES-D) and 0.99 (Pittsburgh Sleep Quality Index [20], PSQI, ‘hours of sleep’). Appraisal of either platform was either good or very good across multiple indicators, comprising quality of instructions, quality of presentation, usability, and general satisfaction with the tool. For example, at first administration ‘general satisfaction’ had the highest score (82 % endorsed the top rank) on the computer platform, whilst ‘instructions’ had the lowest score on the same platform (68 %). However, appraisal ratings between platforms did not differ either at first administration (between independent groups of respondents, n = 66) or when completed at different times (cross-over design only, n = 35). Participants were slower on computer than on paper (Wilcoxon rank-sum test *P* value = 0.01), with median completion time of 12 min (interquartile range, IQR 11–17) and 10 min (IQR 8–14), respectively. Participants filling out questionnaires on a computer had a fourfold data entry correction rate of those filling out the questionnaires on paper at first administration (negative binomial regression incidence rate: 4.2, 95 % confidence interval: 1.6–10.8, *P* value < 0.01), after accounting for sex, age, and computer literacy. The large majority of participants of the cross-over study reported feeling less nervous when operating on computer than on paper (n = 32, 91 %). The majority of participants also reported that in the future they would favour CQ (n = 20, 57 %) versus PQ (n = 2, 6 %), while 13 (37 %) had no preference. By acknowledging major advantages (e.g. accurate data immediately available) and minor limitations (e.g. longer completion time) of the computer-assisted questionnaires, further module improvements were implemented and larger fonts adopted to enhance module visualization. While participants are given the option to skip an entire questionnaire, should they feel uncomfortable with it, the hierarchical structure of the questions is controlled to preserve data consistency and limit missing values.

### Screening interviews and clinical assessment

#### Questionnaires and interview structure

To guarantee the maximal comparability of CHRIS data to other large epidemiological studies, the PhenX toolkit was chosen for questionnaire and phenotype standardization [21]. The questionnaires were grouped in two parts: one filled in with the help of trained interviewers and one self-administered. The list of questionnaires is reported in Tables 2 and 3. A description of the main questionnaires is given in the *clinical assessment* section below, in the context of each specific discipline. Each interview includes the assessment of parental and grandparental information and number of first-degree relatives. This information allows to reconstruct complete family trees up to five generations and opens the possibility of future extensive pedigree reconstruction through interrogation of population records. Upon consent, the interview is recorded for later quality control.

**Table 2:** Clinical domains covered by the CHRIS study. Classes have been adapted by the International Statistical Classification of Diseases and Related Health Problems 10th Revision (ICD-10). Relevant diagnostic codes are reported in parenthesis when matching the assessment modality

Domain	Field (ICD-10 code)	Mode of assessment (source), questionnaire; instrumental assessment
Diseases of the circulatory system	Myocardial infarction; heart failure; cardiac arrhythmias (I20-25)	CAPI (PhenX); 10″ and 20′ ECG
	Essential hypertension (I10)	CAPI (PhenX); blood pressure measurement
	Stroke (I64); including transient cerebral ischemic attack (G45)	CAPI Jackson Heart Study Screening Questionnaire [70]
	Peripheral vascular disease (I73); pulmonary embolism (I26); deep venous thrombosis	CAPI (PhenX)
Endocrine, nutritional and metabolic diseases	Diabetes mellitus (E10-14)	CAPI KORA study [71]; serum glucose, plasma HbA1c
	Disorders of thyroid gland (E00-07)	CAPI; serum TSH, FT4, and FT3
	Obesity and localized adiposity (E65-66)	Body Mass Index, Bioimpedance
Diseases of the genitourinary system	Renal insufficiency (N00-19)	CAPI (PhenX); serum creatinine, urinary albumin-to-creatinine ratio
Diseases of the nervous system: extrapyramidal and movement disorders	Parkinson’s disease (G20)	CAPI [17]
	Essential Tremor (G25.0)	Digitalized spiralography
	Restless Leg Syndrome (G25.8)	CASI RLS Diagnosis and Rating Scale [72]
Diseases of the nervous system: other	Migraine (G43)	CASI Int’l Classif. Headache Disorders (ICHD-II)
	Sleep disorders (sleep quality and behavior) (G47); chronotype (G47.2)	CASI Pittsburgh Sleep Quality Index (PSQI) [20], R.E.M. Sleep Behavior Questionnaire (RBDQ) [73], Munich Chronotype Questionnaire (MCTQ) [28]
	Pain (R52.0) including pain sensitivity	CAPI Pain Sensitivity Questionnaire [27]; pressure and tension gauge algometer
	Chronic pain	CAPI Chronic pain questionnaire
Psychiatric disorders	Anxiety (F40-41)	CASI State-Trait Anxiety Inventory (STAI Y-2) [19]
	Depression (F32-33)	CASI Center for Epidemiologic Studies of the National Institute of Mental Health Depression scale (CES-D) [18]
	Hypomania and mood (F30-39)	Paper-based, self-administered; MINI International Neuropsychiatric Interview (M.I.N.I.) [32]; HCL-32 questionnaire for Energy, Activity and Mood [35]
Cognition and autonomic function	Cognition impairment	CAPI Mini-Mental State Examination (MMSE) [30]
	Autonomic dysfunction	CASI Composite Autonomic Symptom Score (COMPASS 31) [31]
	Disturbances of smell (R43)	Sniffin’ sticks [45]

*CAPI* computer assisted personal interview, *CASI* computer assisted self interview, *SQ* screening questionnaire, *PhenX* based on PhenXToolkit standards (https://www.phenxtoolkit.org/index.php)

**Table 3:** Personal information, life-style, and exposures

Name of the questionnaire (acronym)	Administration mode/method to collect the information (source, reference)	Semi-quantitative outcome
Personal and family information (pedigree reconstruction)	Computer-assisted interviewer-administered questionnaire	NO
Medication, last 7 days (medication bag)	Electronic optical scan of medication boxes	NO
Birth/early-life exposures	Computer-assisted interviewer-administered questionnaire (KORA study) [71]	NO
Food Frequency Questionnaire (including alcohol consumption)	Home-based, paper-based self-administered questionnaire (GA^2^LEN study) [56]	Nutrient intake
Smoking habits	Computer-assisted interviewer-administered questionnaire (EC Respiratory Health Survey II) [55]	Life-course smoking burden (pack-years)
Physical activity	Computer-assisted self-administered questionnaire (Int’l Physical Activity Q) [54]	Metabolic Equivalent of Task (MET)
Environmental factors	Postal address for geo-reference	NO
Occupation	Computer-assisted interviewer-administered questionnaire	NO

#### Cardiovascular health

Screening questionnaires listed in Table 2 are used to capture information about different circulatory system diseases (ICD X codes I00-I99). Diagnostic groups include ischemic heart diseases (including angina pectoris, myocardial infarction), arrhythmic diseases (such as atrial fibrillation, sudden cardiac arrest), and other cardio- and cerebrovascular diseases including atherosclerosis, hypertension, stroke, transient cerebral ischemic attacks, heart failure, pulmonary embolism, circulatory insufficiency, deep venous thrombosis, myocarditis, and cardiomyopathy. Information about the implantation of a pacemaker or an implantable cardioverter defibrillator and the presence of congenital heart malformations is also collected. Biochemical measures taken as potentially reflecting cardiovascular health include CRP, homocysteine, antithrombin III antibodies, fibrinogen and blood clotting times (PT and aPTT) as well as blood counts and cell volumes, and main categories of lipids such as total cholesterol, HDL and LDL forms of cholesterol and triglycerides. Given the strong links with CVD and diabetes, renal function is assessed using the estimated glomerular filtration rate based on serum creatinine, and kidney damage is investigated using the urinary albumin-to-creatinine ratio. Electrolyte homeostasis is assessed by serum magnesium, sodium, potassium, calcium, chloride, and phosphorous, some of which are strongly regulated by the kidney and whose variations impact blood pressure regulation. History and presence of any kidney disease and doctor-diagnosed renal insufficiency are assessed through interview.

#### Metabolic and endocrine health

Body mass index (BMI), fat percentage, visceral fat, and subcutaneous fat are assessed through a body composition monitor (OMRON BF508). Participants are interviewed for doctor-diagnosed diabetes, and fasting serum glucose and glycated hemoglobin (HbA1c) are measured. Since thyroid diseases are the third leading cause of hospitalization in South Tyrol, after hypertension and cardiopathies, thyroid dysfunctions are assessed by means of detailed interviewer-administered questionnaire and by serum thyroid-stimulating hormone (TSH), supported by free thyroxine (FT4) and free triiodothyronine (FT3) in case of abnormal TSH levels. Anti-TPO antibody measurements for thyroid autoimmune disorders are measured as well.

#### Movement disorders

Movement disorders are assessed through screening questionnaires as detailed in Table 2. Among them, essential tremor (ET) is a major focus in the study as it is the commonest type of movement disorder in South Tyrolean adults [10]. It is related to other neurodegenerative conditions, such as Parkinson’s disease. Clinical diagnosis of ET is usually performed by a neurologist, and its severity is assessed through standard rating scales such as the Fahn–Tolosa–Marin Tremor Rating Scale (TRS) [22] or through the quantification of tremor, for instance by the visual assessment of a spiral drawn on paper [23]. However, visual assessment is prone to subjectivity. Clinical assessment often lacks sensitivity to capture small, albeit relevant manifestations of tremor, making it difficult to assess the prodromal phase and progression of the disease. Validity and reliability of clinical and visual assessment of tremor represent an actual challenge, limiting comparability both within and between studies. Digitized spiral analysis (DSA) is performed by drawing a spiral on a digital tablet connected to a computer. Each participant draws six spirals on top of a guide, starting with participant’s preferred hand and alternating between hands after each drawing. Data from the spiral recordings is extrapolated in three spatial dimensions (lateral, longitudinal and vertical) sampled at a rate of 130 Hz. After a semi-automated data cleaning procedure, several indicators are derived from the drawings in the spatial–temporal dimensions, including speed, pressure, acceleration, tremor amplitude, tremor frequency, and tremor direction. The testing hypothesis is that a combination of multiple metrics may help classify subjects according to different patterns of their drawings, which may ultimately link to different types of tremor symptomatology. To the best of our knowledge, this is the largest population-based study to date that has been collecting digital recordings on a spiral. This peculiarity will allow deriving normative range values for these metrics at a population level and relating them to the population characteristics.

#### Chronic pain and pain sensitivity

Chronic pain affects a large proportion of the adult population and represents a major impediment to the physical functioning of individuals [24]. An altered sensitivity to painful stimuli is also related to chronic pain, although the direction of this association is unclear [25, 26]. Both chronic pain and pain sensitivity are measured in the CHRIS study. Chronic pain is assessed through a module in the interview questionnaire, which specifically asks for the duration, intensity and frequency of any pain affecting the musculoskeletal system, in particular back and joints. Pain sensitivity is measured both experimentally and through a questionnaire. The pressure pain threshold (PPT) experimentally measures the level of pressure (kg/cm^2^) at which pain is first perceived during a mechanical stimulus of increasing force. In our case, the pressure is applied vertically on the left index finger of the participant through a hand-held gauge algometer by a trained operator. The procedure is stopped as soon as the participant reports some level of pain. The pressure measured by the algometer at that point represents the individual’s PPT. The pain sensitivity questionnaire [27] (PSQ) is made of 14 items, relating to common daily life situations of mild or moderate painful experience. The respondent reports the imagined intensity of pain for each situation on a scale from 0 (no pain) to 10 (worst imaginable pain). The average of 14 items represents the PSQ-total score and measures the individual overall pain intensity rating. Two subscores are also derived, each by averaging a selection of 7 separate items representing mild painful situations (PSQ-minor) and moderate painful situations (PSQ-moderate), respectively.

#### Sleep and chronotype

Insomnia affects 7 % of the European population [11] and can be present as a primary condition or be related to medical conditions, such as endocrine, neurologic and psychiatric diseases. In particular, discerning whether poor sleep quality is prodromal to onset of mood disorders or vice versa is an active area of research, and identifying common risk factors underlying low quality sleep and psychopathology may lead to new therapeutic targets for both disorders. In CHRIS, sleep quality is measured using the PSQI questionnaire [20]. The Munich Chronotype Questionnaire [28] was also implemented to gain a better understanding of the underlying complexity and individual differences of the biological clock, as shown in everyday behavior. Finally, the rapid eye movement (REM) sleep behavior disorder (RBD) is also assessed as it is often associated with other neurological conditions, such as Lewy body dementia, Parkinson’s disease or multiple system atrophy [29].

#### Cognitive function

Cognitive function is evaluated by the Mini-Mental State Examination (MMSE) [30], a 30-item screening questionnaire assessing different cognitive domains, such as memory, orientation to space and time, and language ability.

#### Autonomic function

The Composite Autonomic Symptom Score (COMPASS 31) [31], a self-administered questionnaire on autonomic dysfunction, is used to assess the presence of autonomic symptoms. It consists of 31 questions about autonomic symptoms in 6 different domains: orthostatic intolerance, vasomotor dysfunction, secretomotor dysfunction, gastrointestinal dysfunction, combining gastroparesis, diarrhea and constipation, bladder dysfunction and pupillomotor dysfunction.

#### Psychiatric health

Depression is assessed through the CES-D questionnaire [18] (Table 2). Anxiety is investigated by using the State-Trait Anxiety Inventory (STAI Y2) [19], a 40-question questionnaire which allows to assess both state and trait anxiety. On a subsample of ~3000 participants, a deeper psychiatric assessment was introduced that encompasses major depression episodes as well as manic and hypomanic episodes [32]. These participants are also assessed for perceived stress [33], childhood trauma [34], mood [35], life-orientation [36], and family history of psychiatric disorders. All interviews are self-administered.

#### Phenotyping of multi-systemic functions

The screening described above spans over several domains. Some of these domains share common pathophysiological mechanisms which can be assessed through additional instrumental or molecular phenotyping, as described below.

*ECG and continuous non*-*invasive arterial pressure measurement* A 10-s and a 20-min 12-lead ECG are recorded using a PC-ECG-System Custo 200 (Customed) workstation with a sampling rate of 1000 Hz. Participants are asked to remain in supine position and silent during the procedure. In addition to classical indicators of heart electrical conduction, such as PR, QRS and QT duration, the 20-min ECG allows the calculation of parameters that reflect the heart rate variability (HRV) [37], which is defined as the temporal variation between consecutive heart beats. HRV reflects the continuous interaction between neural modulatory mechanisms and the sinoatrial node rhythmicity. ECG measurement of HRV has already been described as an important tool to assess autonomic function [38] and to predict cardiovascular events in association with ageing [39], type 2 diabetes [40], metabolic syndrome [41] and other cardiovascular risk factors [42]. It has been recently demonstrated that the deceleration capacity [43], defined as the integral measure of all deceleration-related oscillations, strictly depends on the autonomous nervous system activity and is mainly an index of vagal activation. Furthermore, the periodic repolarization dynamic (PRD) is a parameter describing low frequency rhythmic modulation of repolarization that can be used to specifically assess the sympathetic effect on repolarization [43]. In a sample of 3000 participants, continuous non-invasive arterial blood pressure (BP) is measured and completely synchronized with the 20-min ECG. Such a simultaneous recording allows estimation of the post-extrasystolic BP potentiation (PESP), defined as the pulse wave augmentation in BP after a suitable ventricular premature complex [44]. PESP has been recently shown to strongly predict post infarction mortality. Its usefulness as a predictor of adverse cardiovascular outcome in the general population deserves validation.

*Olfaction assessment* A smell test is assessed with Sniffin’ sticks (Burghardt Medizintechnik, Wedel, Germany) [45]. A total of 16 odors related to common daily life experience have to be recognized from a list of four choices for each odor. Hyposmia, or olfactory dysfunction, is a prodromal symptom for Parkinson’s disease which often anticipates motor symptoms [46] and it has been shown to predict Alzheimer dementia as well [47]. Reduced smell ability was shown to discriminate between multiple system atrophy and pure autonomic failure [48], suggesting a possible role of its assessment in the differential diagnosis among dysautonomic syndromes. Moreover, a vast literature correlates smell dysfunction with schizophrenia [49].

*Metabolomics of energy metabolism* Energy metabolism and mitochondria in general are increasingly being linked to ageing [50, 51] and diseases that present themselves in mid to later stages of life [52, 53]. A systematic literature assessment of metabolites that capture mitochondrial function, with emphasis on metabolites that are directly or indirectly related to energy metabolism was undertaken. As a result, about 70 metabolites were identified with a broad spectrum of chemical properties including, but not limited to, amino acids, heterocyclic compounds, organic acids and mono-phosphate nucleotides. Plasma levels of the identified metabolites are being measured by liquid chromatography–tandem mass spectrometry (LC–MS). In addition to this hypothesis-driven investigation, untargeted metabolomics analysis in a subset of the participants will also be performed to identify novel metabolites.

#### Life-style and environmental exposures

As detailed in Table 3, physical activity, food intake including alcohol consumption, smoking habits, and early life exposures are collected by means of computer-assisted self-and interviewer-administered questionnaires. Some of the questionnaires allow quantification of exposures as either quantitative variables or semi-quantitative scores, which increases the statistical power to detect phenotype–environment associations or gene–environment interactions compared with dichotomous exposures. This is the case for the International Physical Activity Questionnaires [54] (IPAQ), from which the Metabolic Equivalent of Task (MET) can be derived, the European Community Respiratory Health Survey (ECRHS) II smoking questionnaire from which life-course smoking intake in terms of pack-years is derived [55], and the GA^2^LEN food frequency questionnaire (FFQ) from which an estimation of the nutrient intake [56] can be obtained. In the case of the GA^2^LEN FFQ, a paper-based format could not be avoided. The 20-page FFQ includes 229 items and requires ~25 min for completion. To limit the time spent at the study center, the questionnaire is mailed to the participants’ home at the time when the appointment is set up. At the study center, the questionnaire is visually inspected by a study assistant. In case of missing responses, the participant is asked to fill in the missing items. The questionnaire is then processed by an automatic optical mark recognition (OMR) system: all pages are processed with a high throughput scanner and data stored in our central PostgreSQL database system. The deployed OMR system, SDAPS (http://sdaps.org), has been tailored to our study by in-house development. Correct assignment of collected information to participant is guaranteed by use of individual participants’ barcode reported on each page. Correct sequence of data entered is guaranteed by an additional page specific barcode. Based on the first 1000 participants, 100 % of the questionnaires was returned and 81 % had no missing items.

## Results

In 2015, we froze the first data release which included 4979 participants (male:female ratio = 44 %:56 %) recruited until 15 July 2014. Participants’ characteristics by gender are given in Table 4. On average, subjects were 46.2 years old (SD = 16.4) with no difference between sexes. Ninety-four percent of subjects was born in South Tyrol. A diploma from a vocational school was the most common educational level attained, with females having a higher educational level than males. Eighteen percent males and 5.9 % females reported daily alcohol consumption. Current smokers were 19.7 % of males and 16.7 % of females (*P* < 0.01). The majority of the sample was classified as doing high physical activity. Doctor-diagnosed hypertension was reported by 25.1 % males and 21.5 % females (*P* < 0.01) and 60.7 % males and 41.3 % females had a BMI > 25 (*P* < 0.01). When defining diabetes status based on serum glucose and glycated hemoglobin levels (see Table 4 footnote), 4.6 % males and 4.2 % females were classified as having diabetes, while a pre-diabetic condition was identified in 40.4 % males and 41.7 % females (*P* value 0.55). Finally, females had a slightly higher total cholesterol level than males (*P* < 0.01). Table 5 shows that the age and sex structure of the recruited sample is very similar to that of the general population of the region. An exception is the 75+-year-old group, which is underrepresented in the CHRIS study due to the logistic difficulty of very old people to travel to the study center. However, in general such similar distribution between sample and reference population suggests that the CHRIS study sample will be valuable also for epidemiological investigations. As an additional characterization of this sample, we observed 606 singleton and 4373 related subjects out of the 4979 participants. The related subjects could be connected through 186 pedigrees characterized by 3014 founders, each one with 1–56 descendants (mean 6). Finally, 4570 samples have been fully genotyped and are available for genome-wide association studies. This dataset will constitute the first nucleus for epidemiological investigations and genetic data analysis.

**Table 4:** Characteristics of the 4979 participants included in the first CHRIS data release by gender

Participants’ characteristics^a^	Males N = 2212	Females N = 2767	*P* value^b^
Age (years)—mean (SD)	46.5 (16.4)	45.9 (16.3)	0.22
Self-reported origin—n (%)			
South Tyrol	2087 (94.4)	2591 (93.6)	0.40
European	122 (5.5)	174 (6.3)	
Non-European	3 (0.1)	2 (0.1)	
Education, n = 4976—n (%)			
No formal education or degree	5 (0.2)	7 (0.3)	<0.01
Primary school	223 (10.1)	379 (13.7)	
Lower secondary school	311 (14.1)	401 (14.5)	
Vocational school	1070 (48.4)	985 (35.6)	
Upper secondary school	427 (19.3)	703 (25.4)	
University	176 (7.9)	289 (10.5)	
Alcohol consumption frequency^c^, n = 4974—n (%)			
Never to seldom	489 (22.1)	1389 (50.3)	<0.01
≤1 day/week	684 (30.9)	897 (32.5)	
≥2 days/week	630 (28.5)	313 (11.3)	
Daily	408 (18.5)	164 (5.9)	
Smoking habits, n = 4977—n (%)			
Never smoker	1024 (46.3)	1550 (56.1)	<0.01
Past smoker	752 (34.0)	753 (27.2)	
Current smoker	436 (19.7)	462 (16.7)	
Physical activity, n = 4683—n (%)			
Low	502 (24.2)	626 (24.0)	<0.01
Moderate	507 (24.5)	853 (32.7)	
High	1063 (51.3)	1132 (43.4)	
Blood pressure (BP), n = 4971—mean (SD)			
Systolic BP (mm/Hg)	124.5 (14.5)	115.3 (17.1)	<0.01
Diastolic BP (mm/Hg)	79.3 (9.0)	76.6 (9.5)	<0.01
Elevated blood pressure^d^, n = 4953—n (%)			
No	1647 (74.9)	2163 (78.5)	<0.01
Yes	551 (25.1)	592 (21.5)	
Body-Mass-Index kg/m^2^, n = 4916—n (%)			
<25	856 (39.3)	1607 (58.7)	<0.01
25–30	972 (44.6)	701 (25.6)	
≥30	350 (16.1)	430 (15.7)	
Diabetes status^e^, n = 4967—n (%)			
No	1215 (55.0)	1491 (54.0)	0.55
Prediabetes	891 (40.4)	1151 (41.7)	
Diabetes	102 (4.6)	117 (4.2)	
Serum total cholesterol (mg/dl)—mean (SD)	207.8 (41.7)	213.0 (40.3)	<0.01

^a^Indicated is the actual number of non-missing values when <4979

^b^ *P* values for difference between males and females were obtained from *t* and Fisher’s exact tests for continuous and categorical variables, respectively

^c^Based on the question: “*During the last 12* *months, on average how often have you drunk alcoholic drinks?*”

^d^Based on the question: “*Has a doctor ever said that you have high blood pressure or hypertension?*”

^e^Prediabetes: serum glucose (mg/dl) 100–125 or glycated hemoglobin (HbA1c) (%) 5.7–6.4; diabetes: serum glucose (mg/dl) ≥ 126 or glycated hemoglobin (HbA1c) (%) ≥ 6.5

**Table 5:** Age and sex distribution of CHRIS participants compared to the general population of Vinschgau/Val Venosta

Age	First CHRIS data release			Resident population 2012^a^		
	Males	Females	Total	Males	Females	Overall
	n (%)	n (%)	n (%)	n (%)	n (%)	n (%)
18–29	442 (20.0)	576 (20.8)	1018 (20.5)	2834 (19.9)	2661 (18.7)	5495 (19.3)
30–44	583 (26.4)	739 (26.7)	1322 (26.6)	3864 (27.1)	3659 (25.7)	7523 (26.4)
45–59	708 (32.0)	879 (31.8)	1587 (31.9)	4001 (28.0)	3739 (26.3)	7740 (27.2)
60–74	370 (16.7)	441 (15.9)	811 (16.3)	2340 (16.4)	2358 (16.6)	4698 (16.5)
75+	109 (4.9)	132 (4.8)	241 (4.8)	1238 (8.7)	1803 (12.7)	3041 (10.7)
Overall	2212 (100)	2767 (100)	4979 (100)	14,277 (100)	14,220 (100)	28,497 (100)

^a^Data are referred to the same municipalities included in the study. Data were obtained by the Statistical Institute of the Autonomous Province of South Tyrol (ASTAT): http://www.provincia.bz.it/astat/it/service/dati-online.asp

## Discussion

Biomedical research is witnessing a paradigm shift from sickness to health, where the best way to prevent disease onset is to understand how the whole ageing process works in healthy individuals. For this purpose, longitudinal studies based on the general population are of the greatest relevance. In addition to screening population health through extensive interviews and clinical examinations, the CHRIS study has some distinctive features.

The target population in this restricted area of the Alps is characterized by a rather homogeneous life-style. Previous work from our group demonstrated that the population in this area is stable, with low residential mobility across generations [12, 57, 58] and low inbreeding [59]. We previously observed a low to null impact of shared environmental components on biomarker heritability [13], which suggests homogeneous life-style and environmental conditions. Such a homogeneity would constitute an advantage in mapping causal genetic variants in this geographical area. The collection of non-genetic exposures such as diet, smoking, and physical activity will allow us to assess the extent of variability of life-style in this region and to test candidate gene-environment interactions for biomarker traits of interest and the most prevalent disease outcomes.

The reconstruction of familial information will allow the building of medium sized pedigrees which will facilitate the mapping of inherited variants. With the emphasis currently being on low frequency genetic variants, identified through SNP arrays or next-generation sequencing technology, sampling a large set of the resident population in a small region is advantageous in terms of allowing reliable identification of alleles that may be rare elsewhere but possibly overrepresented in the region. While the level of enrichment of low frequency variants may not be expected to be as large as that of populations undergoing strong genetic isolation [60], it is likely that the CHRIS population sample could show more enrichment than general population studies. The absence of founder effects may keep the effective population size of the study large [61] which, paired with environmental homogeneity, should translate into higher power of detecting genetic associations. The study is planned to recruit about 10,000 subjects, corresponding to roughly 35 % of the entire population in the region. Despite the typical issues of Alpine valleys in respect to traveling time and conditions necessary to reach the study center especially from the more remote villages, we believe this rate is reasonably achievable.

Currently, the genetic epidemiology community is facing the issue of data sharing and harmonization. Data harmonization, pooling, and sharing are beneficial to scientific research provided that data security and privacy are guaranteed [62]. This aspect may be particularly relevant now that rare genetic variants are being considered more frequently, given their functional nature, and data-sharing might become the only way to increase study power. In fact, population-based research is under two opposite ethical pressures: on the one hand it is imperative to protect data privacy and security, on the other hand, an ethically responsible research should aim at maximizing the use of stored data and samples, so as to guarantee the maximal benefit to the community [16]. For this reason, with the aim of maximizing the scientific harvest of the CHRIS study, collaborations that are instrumental to data harmonization within the Italian Hub of Population Biobanks [63] and the Biobank Standardisation and Harmonisation for Research Excellence in the European Union (BioSHaRE) initiative [62, 64] were set up. In particular, the BioSHaRE project framework allows sharing data analysis results without real sharing of the personal-level data, thanks to the new DataSHIELD technology [65].

A relative advantage of a population-based study of limited sample size conducted in a small region might be the quicker turn-around to establish focused research studies nested into the CHRIS study. The strict collaboration with GPs and hospital doctors from the valley’s central hospital made it easier to build up a trust relationship, so that re-invitation of participants for additional deep phenotyping has been proven to be a realistic target. Along the same line, from a biobanking perspective, such a manageable sample size opens up the possibility of creating a very thick layer of phenotypes from the collected biomaterial. Projects are already ongoing that will establish a broad range of molecular phenotypes covering a wide spectrum of metabolites through LC–MS analysis, effectively extending our previous work [14, 66]. In this context, small studies are particularly suitable for gene discovery, given that essential metabolites are generally regulated by a very few genes with large effects [14, 66].

Europe is under an unprecedented ageing phase, which brings the need of a global initiative to prevent morbidities typical of the elderly [67]. A global approach includes health promotion among the most important policy stakeholders. By enrolling a large portion of the total population and directly involving all GPs and municipality councils of the region, the CHRIS study acts as an important player in raising the attention towards healthier ageing. In addition to the unavoidable sensitization which passes through invitation and participation in our screening program, participants are explicitly recommended to visit their reference GPs for interpretation of the clinical examination results. Informative events are planned in collaboration with local charities promoting knowledge and prevention of the diseases, so that CHRIS researchers are involved in health-promoting events which go beyond the communication about the study. Current efforts are being dedicated to create groups of local promoters of healthy ageing studies, which may help in sensitizing the population towards participation and may also have a role in highlighting special population needs so as to orient research towards applied solutions that are closer to the population needs. To propose a concept where health can be shaped together by people and scientists, a general initiative was started, where the representatives of the largest associations of the valley together with GPs and local schools are being involved. This group has the purpose to promote health-related initiatives in the valley, increase the level of attention towards health-related topics, and hopefully create a philanthropy culture which can not only support, but foster scientific biomedical research in the region.

In a time when very large biobanks have been established [68], strict scientist-population turnaround may be an advantage for mid-sized studies in order to achieve phenotype coverage of the target population, precision and homogeneity of the phenotype measurements, and support from the study participants themselves to expand the study into novel areas of biomedicine. On these premises, the CHRIS study will serve as foundation for a larger biomedical research effort aimed at uncovering genetics and molecular markers that can be predictive of individuals’ ageing trajectories as well as serve as potential therapeutic targets in disease progression control [69].
